# The Isoelectric Region of Proteins: A Systematic Analysis

**DOI:** 10.1371/journal.pone.0010546

**Published:** 2010-05-07

**Authors:** Michael Widmann, Peter Trodler, Jürgen Pleiss

**Affiliations:** Institute of Technical Biochemistry, University of Stuttgart, Stuttgart, Germany; Griffith University, Australia

## Abstract

**Background:**

Binding of proteins in ion exchange chromatography is dominated by electrostatic interactions and can be tuned by adjusting pH and ionic strength of the solvent. Therefore, the isoelectric region (IER), the pH region of almost zero charge near the pI, has been used to predict the binding properties of proteins.

**Principal findings:**

Usually the IER is small and binding and elution is carried out at pH values near to the pI. However, some proteins with an extended IER have been shown to bind and elute far away from its pI. To analyze factors that mediate the size of the IER and to identify proteins with an extended IER, two protein families consisting of more than 7000 proteins were systematically investigated. Most proteins were found to have a small IER and thus are expected to bind or elute near to their pI, while only a small fraction of less than 2% had a large IER.

**Conclusions:**

Only four factors, the number of histidines, the pI, the number of titratable amino acids and the ratio of acidic to basic residues, are sufficient to reliably classify proteins by their IER based on their sequence only, and thus to predict their binding and elution behaviour in ion exchange chromatography.

## Introduction

Ion exchange chromatography (IEC) is a widely applied method in protein purification. It is well established, efficient, and applicable to large scale purification [1], [2]. Protein binding in IEC is primarily determined by electrostatic interactions between the charge of the protein and the charged stationary phase [3], [4], [5]. As a consequence, optimal pH values for binding to or elution from an ion exchange column can be predicted by the isoelectric point (pI) for many proteins [6] with loading pHs about 0.5–1 pH units above or below the pI of the respective protein [7], [8]. However, it has been shown that for some proteins the pI is not predictive, but binding to or elution from the column only occurs for pH values far from the pI of the protein [6], [9]. A detailed investigation of pH values at which bound proteins eluted from an anion exchange chromatography column were performed using a pH gradient as the method of elution. It demonstrated that for proteins with pI values between 6 and 8, the elution occurred at pH values considerably higher than their pI. Proteins with a pI lower than 6 or higher than 8 however eluted at pH values close to their pI [6]. The unique behaviour of proteins with a pI between 6 and 8 was explained by the observation that their titration curves had a broad region of almost zero charge near their pI which extended over several pH units [6]. In this work, we term this region the isoelectric region (IER). A large IER has also been shown to influence the binding of proteins to ion exchange columns which has been demonstrated for the lipase B from *Candida antarctica* [9]. The purification of this protein by ion exchange chromatography had not been achieved before. Only by taking the large IER into account and substantially lowering the binding pH to 3, which is 3 pH units lower than the pI of the protein and beyond the proteins IER, a successful binding to a cation exchange column was achieved. Therefore, a large IER is expected to lead to differences between the pI of a protein and its pH of binding to or elution from a column. Two factors which can be easily extracted from the protein sequence have been suggested to determine a large IER: a pI of the protein between 6 and 8 [6], and a low number of histidines [9], since histidine is the only titratable residue in the pH region between 5 and 9.

In this work, we investigated these factors by a systematic analysis of two protein families, the α/β hydrolase family with more than 4600 proteins and the medium-chain dehydrogenase/reductase protein family with more than 2600 proteins, based on the Lipase Engineering Database [10] and the Medium-Chain Dehydrogenase/Reductase Engineering Database [11], respectively. Both protein families had previously been integrated in our data warehouse system for protein families DWARF [12]. The members of each protein family share a similar structure but have highly diverse sequences. In addition, the results were compared to a set of 5000 randomly generated protein sequences. The frequency of proteins with a large IER and the influence of the previously suggested factors like the number of histidines and the pI on the IER were investigated in order to establish a set of factors with a correlation to the IER. The ratio of acidic and basic amino acids R was included as a factor for this analysis since it had been previously shown to correlate with the pI [13]. These factors could be used to change the IER by protein engineering in order to facilitate the purification process by ion exchange chromatography methods. To allow for a direct access to data on the isoelectric point and the size of the IER, these values were pre-calculated and integrated in our database model.

## Results

### Isoelectric region

**Figure 1:**
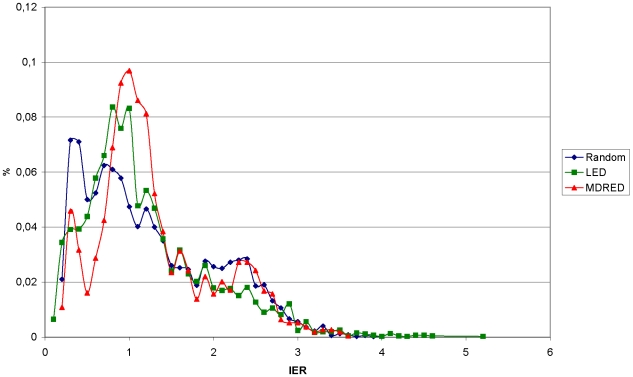
Comparison of protein distribution according to IER size. Protein numbers are displayed as percentages. Results from the α/β hydrolase database (LED) and the medium-chain dehydrogenase/reductase protein family (MDRED) are overlaid with results from the random set.

The size of the region of very low total charge (larger than −3 and smaller than 3) near the pI of a protein differs significantly between proteins and was termed the isoelectric region (IER) in this work. Proteins with a small IER are expected to bind to or elute from an ion exchange column at a pH value close to their pI because their total charge sensitively depends on the pH at values close to their pI. Proteins with a large IER, however, are expected to bind to or elute from an ion exchange column at pH values that are noticeably higher or lower than their pI due to the elongated area of almost zero charge in proximity to the pI. To determine the number of proteins with a large IER, the IER of 4652 sequences from the α/β hydrolases family and 2683 sequences from the medium-chain dehydrogenase/reductase protein family were evaluated and systematically analyzed and compared to 5000 random sequences. The calculated IER ranged from 0.1 to 5.2. The proteins were divided into 2 groups depending on their IER: proteins with a small IER (0.1≤IER<3) and proteins with a large IER (3≤IER). For both protein families and the random set the majority of proteins (98%) belonged to the first group with a small IER. The distribution of proteins in regard to the IER was found to be identical for all three protein sets and only a small minority of proteins in each set (2%) belonged to the group with a large IER (Figure 1). Only a few protein families constituted the group with a large IER. For the α/β hydrolase family, 40% of proteins with a large IER were members of the ‘cutinase’, ‘antigen 85’, or ‘carboxylesterase’ families. For the medium-chain dehydrogenase/reductase protein family, 70% of all proteins with a large IER belonged to the ‘YADH’ or the ‘QOR like’ families. However, the majority of proteins in these protein families also had a small IER.

### Factors influencing the IER

Several factors were investigated for their correlation with the size of the IER. The goal was to identify a single factor or a combination of factors which showed a strong correlation with the IER. Factors that were considered for this analysis were the number of histidines, the pI of the protein, and the related ratio R between acidic and basic amino acids. These factors were chosen since they had already been shown or suggested to influence a proteins IER.

The number of histidines and the size of the IER were determined for every protein. For proteins with an identical number of histidines, the mean IER and its standard deviation were calculated. If only 2 or less proteins had the same number of histidines they were excluded from the analysis. The number of histidines showed only a weak correlation to the size of the IER for the α/β hydrolase and medium-chain dehydrogenase/reductase families, and no correlation for the random set (Figure S1). Furthermore, proteins with the same number of histidines showed considerable differences in the size of the IER indicated by large standard deviations (e.g. ±1.5 for proteins with 2 histidines). The number of histidines, independent of other factors, was therefore shown to be an inadequate factor for the prediction of the IER.

The other previously suggested factor to be indicative for proteins with a large IER was a pI value between 6 and 8. Therefore the pI was determined for each protein and the proteins were divided into two groups based on their pI. One group consisted of proteins with a pI between 6 and 8, the other group of proteins with a pI lower than 6 or higher than 8. Proteins with a pI between 6 and 8 were shown to have a higher percentage of proteins with a large IER (3–5%) than proteins with a pI below 6 or above 8 (1%). This distribution was observed for both protein families and the random set.

The analysis of the correlation between the number of histidines and the size of the IER was repeated for the two protein groups that were assigned based on pI. Now, a strong dependence between the number of histidines and the size of the IER was observed for proteins with pI values between 6 and 8 for both protein families and the random set (Figure S2). Proteins with the same number of histidines had a similar IER as indicated by small standard deviations and showed a steady decrease of their IER with an increasing number of histidines. For proteins with a pI lower than 6 or higher than 8 only a very weak correlation to the number of histidines could be observed and proteins in this group generally had a small IER. However, a few proteins in this group still showed a large IER indicated by the large standard deviations in this set.

To find a factor that identifies proteins with a small IER, independently of the number of histidines, the ratio R of acidic and basic amino acids was combined with the number of titratable residues, because we observed that all proteins with a large IER showed a balanced ratio R of acidic and basic amino acids and a low number of titratable residues, in contrast to the majority of proteins with a small IER. These two properties were combined into a new balance factor B by multiplying the number of titratable residues by |ln R| (Material and Methods). Analogous to the classification of proteins by their pI, the factor B was used to separate the proteins into two groups. Both groups were then evaluated for a correlation between the number of histidines and the size of the IER.

**Figure 2:**
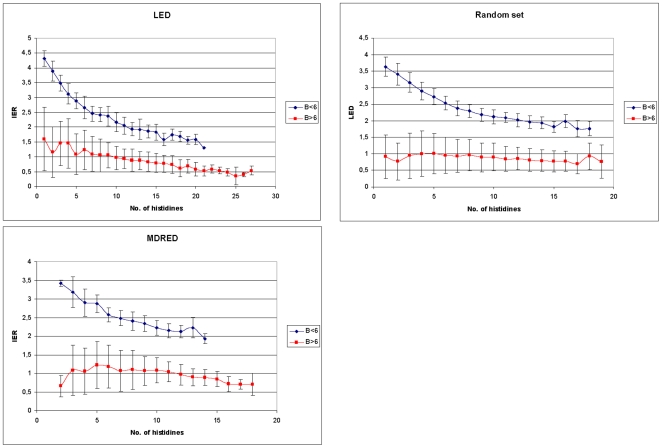
Number of histidines and isoelectric region (IER) for protein family groups. For proteins with the same number of histidines, the median IERs are plotted against the number of histidines. Proteins with (B≤6) are depicted in blue, proteins with (B>6) are depicted in red.

According to this evaluation, a threshold of 6 was selected for the factor B which yielded the best separation for both groups in regard to the correlation between the IER and the number of histidines. For proteins with B≤6, a strong dependence of the IER on the number of histidines was observed (Figure 2). For the α/β hydrolase family, the average IER for proteins with one histidine was 4.3. It decreased to 2.6 for proteins with 6 histidines, and to 1.6 for proteins with 18 histidines. This was similar to the average IER from the medium-chain dehydrogenase/reductase protein family which also showed an average IER of 2.6 for proteins with 6 histidines. The set of random sequences showed an average IER of 3.4 for proteins with two histidines and an IER of 2.5 for proteins with 6 histidines. In contrast, all proteins with B>6 had a small IER of less than 3 (Table 1), showed no correlation of the IER with the number of histidines and displayed a median IER of 1.6 or less for proteins with the same number of histidines (Figure 2).

**Table 1:** Distribution of proteins according to IER size in dependency of B.

Protein family	(IER ≥3)	
	B < = 6	B >6
α/β hydrolases	13%	0%
dehydrogenases/reductases	10%	0%
Random set	8%	0%

### Database integration

**Figure 3:**
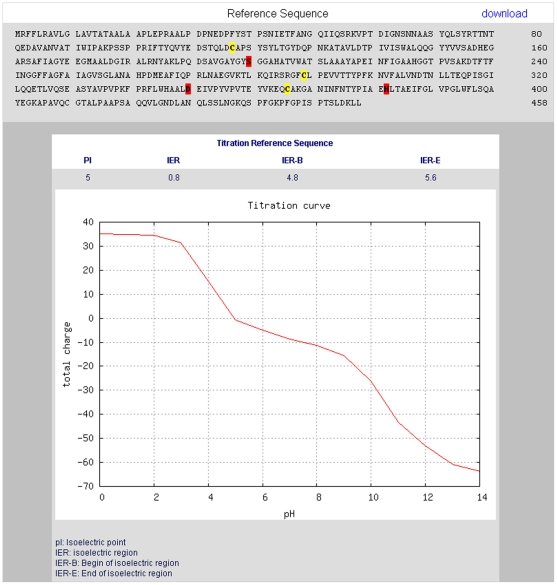
Web interface of the LED with the electrostatic properties feature. Protein total charge is displayed for each pH value from 0–14 in graphical form. Graphical representations of titration curves are generated by GNUPLOT. The isoelectric point and the size of the isoelectric region are given in tabular form.

The values for the pI, IER and the charge of the protein for pH values 0–14 were calculated and integrated in the database model of the Lipase Engineering Database (LED). The pI and the titration curve are directly accessible via the web interface and are displayed in tabular as well as in graphical form for the selected sequence (Figure 3). In addition, the size of the isoelectric region (IER) is calculated and displayed. The LED is accessible by a web interface at http://www.led.uni-stuttgart.de.

## Discussion

The isoelectric region (IER) of a protein is known to have a considerable influence on the binding to or elution from an ion exchange column [6], [9]. This influence is based on the size of the region of almost zero charge near the pI of the protein. For proteins with a small IER, the optimal pH for binding or elution can usually be predicted by their pI. While this prediction often matches experimental results reasonably well, other factors besides the net charge can influence the binding behaviour of proteins to ion exchange columns. This includes the surface charge distribution [3], protein hydrophobicity [14], [15], van der Waals interactions [16], and choice of the adsorbent materials [5], [17]. It has also been shown that not only the amino acid composition of a protein but also its subsequent modification can influence the elution behaviour of proteins in ion exchange chromatography, e.g. by glycosylation which might lead to the shielding of surface charges [18]. For proteins with a large IER however, the net charge in combination with the IER has been shown to be the major factor that influences binding to [9] or elution from [6] an ion exchange column. While the pI is a widely used parameter for the estimation of the electrostatic interactions of proteins, the prediction of the IER is frequently neglected. This can be explained by the small number (2%) of proteins having a large IER. However, for these proteins the importance of the IER has been demonstrated and should be taken into consideration in addition to the pI. The size of the IER can be easily determined from a calculated or experimentally determined titration curve.

In order to understand the molecular basis of a small or large IER, factors that correlate with the size of the IER were identified and analysed. This included previously suggested factors like a pI between 6 and 8 [6] or a low number of histidines [9]. We could show that neither of these factors was correlating with the size of the IER on its own. However, by combining two factors into the balance factor B, proteins which showed a correlation of the IER to the number of histidines were identified. Proteins with a value of B less than 6 had a large IER if they included only a small number of histidines, while their IER decreased with increasing number of histidines. For these proteins, the number of histidines is not only a good indicator of the size of the pI, but histidine would also be the major target of engineering a variant with a changed IER. The study also showed that for many proteins the size of the IER sensitively depends on the number and ratio of charged amino acids. Even a small number of amino acid exchanges in protein mutants or isoforms may therefore have a large impact on the optimal pH of binding to an ion exchange column and other charged surfaces.

The integration of the isoelectric point and the size of the IER in our database model of the LED furthermore allows for a direct access to these values and a visualization of the titration curve for each protein in the database.

## Materials and Methods

### Titration curve calculation

Protein sequences were taken from the Lipase Engineering Database [10] and the Medium-Chain Dehydrogenase/Reductase Engineering Database [11]. Sequences with 100% sequence identity and fragments with a length of less than 160 amino acids were excluded, resulting in a total of 4652 sequences from the α/β hydrolase family and 2683 sequences from the dehydrogenase/reductase protein family A set of 5000 random sequences was generated using frequencies for the titratable amino acids from [19] (Table S1). The distribution of titratable amino acids was similar to the distribution found in the α/β hydrolase and dehydrogenase/reductase protein families (Table S2, Table S3). The random set had a defined protein size range between 250–450 amino acids, similar to the size distribution of the dehydrogenase/reductase protein family. Protein charges were calculated using the module “pICalculator” from the Bioperl toolkit [20]. 6 titratable amino acids were included: aspartate (Asp), glutamate (Glu), histidine (His), tyrosine (Tyr), lysine (Lys), and arginine (Arg); pK_a_ values were assigned as described previously in the Emboss pKa set [21]: 3.9, 4.1, 6.5, 10.1, 10.8, and 12.5, respectively. The N- and C- termini had a pK_a_ of 8.6 and 3.6 respectively. Cysteine (Cys) was treated as a nontitratable residue because sequence-based methods are unable to distinguish between free cysteines and cysteines that are part of disulfide bridges. For 112 α/β hydrolases with experimentally determined structures, at least 65% of all cysteines were found to be part of disulfide bridges (data not shown). This number is supposed to be even higher because not all disulfide bridges are properly annotated in the structure entries. Previously it was found that 91% of all cysteines were part of disulfide bridges in over 50 analyzed proteins [13].

In order to validate the accuracy of predictions calculated with the Emboss pKa set, a comparison of this set, a more recent pKa set [22], and a structure based method (PDB2PQR/PROPKA [23]) was performed. 25 proteins with resolved crystal structures were randomly chosen from the data set, and the amino acid sequences used for all calculations were extracted from the crystal structure file. For pH values between 1 to 14, the total charge of the proteins was calculated as the sum of the partial charges of each titratable group. The comparison demonstrated that for the sequence based methods the deviation between the predicted IER and pI values were less than 0.3 and 0.4, respectively (Table S4). The deviation between the Emboss pKa set and the structure based approach using PDB2PQR/PROPKA [23] was less than 0.6 for the IER and 0.8 for the pI (Table S5, Figure S3).

### Ratio between acidic and basic amino acids

Previously, it was shown that the pI of a protein correlates with the ratio R of acidic and basic amino acids:
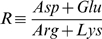
Proteins with a balanced ratio R≈1 showed a high sensitivity of pI to R, while for proteins with an unbalanced ratio the pI was insensitive to R. Previously the ratio R was compared to experimentally determined pI values for 58 proteins [13].

Since a factor R of 1 implies a balance of acidic and basic amino acids, the absolute value of the logarithm of R is a good measurement for the imbalance between acidic and basic amino acids. The introduced balance factor B takes the total number of titratable residues of each protein into account in addition to the distribution of acidic and basic amino acids as represented by |ln R|. The total number of titratable residues was designated as T and is multiplied with |ln R|, resulting in the factor B.
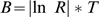



## Supporting Information

Figure S1 Number of histidines and isoelectric region (IER) for each protein family. For proteins with the same number of histidines, the median IERs are plotted against the number of histidines.
(0.03 MB DOC)

Figure S2 Number of histidines and isoelectric region (IER) for each protein family depending on the pI. For proteins with the same number of histidines, the median IERs are plotted against the number of histidines. Proteins with (6< = pI< = 8) are depicted in blue, proteins with (6>pI; pI>8) are depicted in red.
(0.04 MB DOC)

Figure S3 Comparison of sequence to structure based predictions of the IER and the pI Values for the IER and the pI were taken from Table S5.
(0.08 MB DOC)

Table S1 Probabilities of titratable amino acids in percentages used for the creation of random sequences.
(0.03 MB DOC)

Table S2 Distribution of titratable amino acids in percentages for all proteins of the α/β hydrolase family.
(0.03 MB DOC)

Table S3 Distribution of titratable amino acids in percentages for all proteins of the dehydrogenase/reductase family.
(0.03 MB DOC)

Table S4 Comparison of the calculated values for the IER and the pI of 25 proteins. Sequences were extracted from the crystal structure file given for each protein. Values were calculated with the Emboss pKa set used for all calculations in this work and more recent pKa values from (Grimsley et al. 2009).
(0.07 MB DOC)

Table S5 Comparison of the calculated values for the IER and the pI of 25 proteins. Sequences were extracted from the crystal structure file given for each protein. Values were calculated with the Emboss pKa set and compared to the results of a structure based prediction performed with PDB2PQR/PROPKA with the Parse force field.
(0.07 MB DOC)
