# Supplementary material for: The Isoelectric Region of Proteins: A Systematic Analysis
Source: PLoS One. 2010 May 7;5(5):e10546. doi: 10.1371/journal.pone.0010546 (PMC2866324; doi:10.1371/journal.pone.0010546)
Supplement: Figure S1 — Number of histidines and isoelectric region (IER) for each protein family. For proteins with the same number of histidines, the median IERs are plotted against the number of histidines. (0.03 MB DOC) [file pone.0010546.s001.doc]

|  |  |
| --- | --- |
|  |  |

**Figure S1 - Number of histidines and isoelectric region (IER) for each protein family.**

For proteins with the same number of histidines, the median IERs are plotted against the number of histidines.
