# Supplementary material for: The Isoelectric Region of Proteins: A Systematic Analysis
Source: PLoS One. 2010 May 7;5(5):e10546. doi: 10.1371/journal.pone.0010546 (PMC2866324; doi:10.1371/journal.pone.0010546)
Supplement: Figure S2 — Number of histidines and isoelectric region (IER) for each protein family depending on the pI. For proteins with the same number of histidines, the median IERs are plotted against the number of histidines. Proteins with (6< = pI< = 8) are depicted in blue, proteins with (6>pI; pI>8) are depicted in red. (0.04 MB DOC) [file pone.0010546.s002.doc]

|  | |
| --- | --- |
|  |  |

**Figure S2 - Number of histidines and isoelectric region (IER) for each protein family depending on the pI.** For proteins with the same number of histidines, the median IERs are plotted against the number of histidines. Proteins with (6 <= pI <= 8) are depicted in blue, proteins with (6 > pI; pI > 8) are depicted in red.
