# Supplementary material for: The Isoelectric Region of Proteins: A Systematic Analysis
Source: PLoS One. 2010 May 7;5(5):e10546. doi: 10.1371/journal.pone.0010546 (PMC2866324; doi:10.1371/journal.pone.0010546)
Supplement: Figure S3 — Comparison of sequence to structure based predictions of the IER and the pI Values for the IER and the pI were taken from Table S5. (0.08 MB DOC) [file pone.0010546.s003.doc]

| 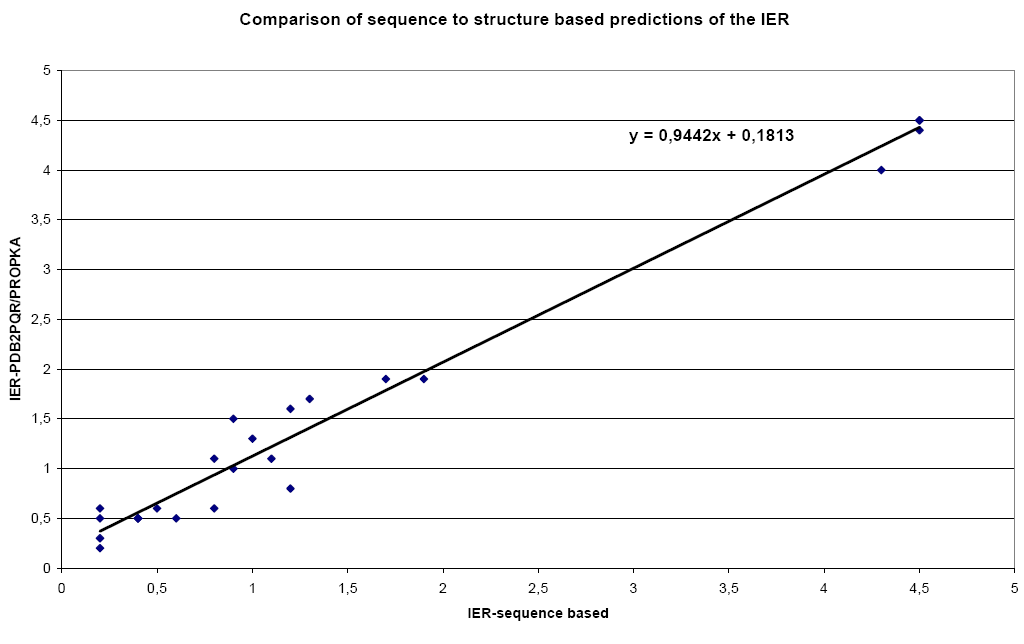 |
| --- |
| 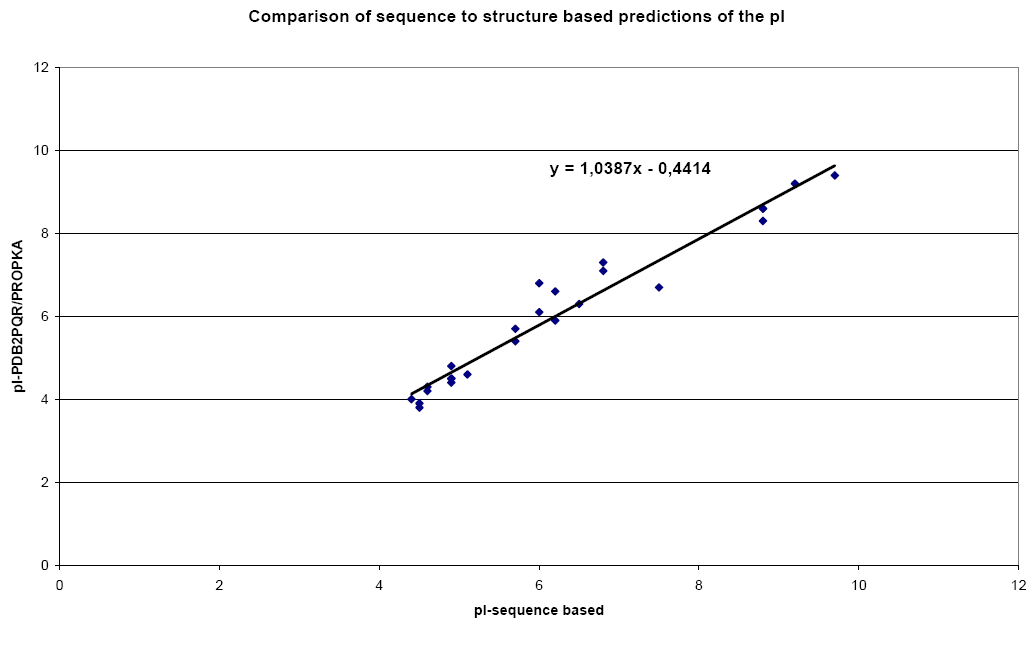 |

**Figure S3 – Comparison of sequence to structure based predictions of the IER and the pI**

Values for the IER and the pI were taken from Table S5.
