# Supplementary material for: The Isoelectric Region of Proteins: A Systematic Analysis
Source: PLoS One. 2010 May 7;5(5):e10546. doi: 10.1371/journal.pone.0010546 (PMC2866324; doi:10.1371/journal.pone.0010546)
Supplement: Table S1 — Probabilities of titratable amino acids in percentages used for the creation of random sequences. (0.03 MB DOC) [file pone.0010546.s004.doc]

**Table S1**

Probabilities of titratable amino acids in percentages used for the creation of random sequences.

| Aspartate (Asp) | 5.2 |
| --- | --- |
| Glutamate (Glu) | 6.2 |
| Histidine (His) | 2.3 |
| Tyrosine (Tyr) | 3.2 |
| Lysine (Lys) | 5.8 |
| Arginine (Arg) | 5.2 |
