# Supplementary material for: The Isoelectric Region of Proteins: A Systematic Analysis
Source: PLoS One. 2010 May 7;5(5):e10546. doi: 10.1371/journal.pone.0010546 (PMC2866324; doi:10.1371/journal.pone.0010546)
Supplement: Table S2 — Distribution of titratable amino acids in percentages for all proteins of the α/β hydrolase family. (0.03 MB DOC) [file pone.0010546.s005.doc]

**Table S2**

Distribution of titratable amino acids in percentages for all proteins of the α/β hydrolase family

| Aspartate (Asp) | 5.6 |
| --- | --- |
| Glutamate (Glu) | 5.2 |
| Histidine (His) | 2.7 |
| Tyrosine (Tyr) | 3.9 |
| Lysine (Lys) | 4.6 |
| Arginine (Arg) | 4.7 |
