# Supplementary material for: The Isoelectric Region of Proteins: A Systematic Analysis
Source: PLoS One. 2010 May 7;5(5):e10546. doi: 10.1371/journal.pone.0010546 (PMC2866324; doi:10.1371/journal.pone.0010546)
Supplement: Table S3 — Distribution of titratable amino acids in percentages for all proteins of the dehydrogenase/reductase family. (0.03 MB DOC) [file pone.0010546.s006.doc]

**Table S3**

Distribution of titratable amino acids in percentages for all proteins of the dehydrogenase/reductase family

| Aspartate (Asp) | 5.4 |
| --- | --- |
| Glutamate (Glu) | 6 |
| Histidine (His) | 2.6 |
| Tyrosine (Tyr) | 2.3 |
| Lysine (Lys) | 5.1 |
| Arginine (Arg) | 4.5 |
