# Supplementary material for: The Isoelectric Region of Proteins: A Systematic Analysis
Source: PLoS One. 2010 May 7;5(5):e10546. doi: 10.1371/journal.pone.0010546 (PMC2866324; doi:10.1371/journal.pone.0010546)
Supplement: Table S4 — Comparison of the calculated values for the IER and the pI of 25 proteins. Sequences were extracted from the crystal structure file given for each protein. Values were calculated with the Emboss pKa set used for all calculations in this work and more recent pKa values from (Grimsley et al. 2009). (0.07 MB DOC) [file pone.0010546.s007.doc]

**Table S4**

Comparison of the calculated values for the IER and the pI of 25 proteins. Sequences were extracted from the crystal structure file given for each protein. Values were calculated with the Emboss pKa set used for all calculations in this work and more recent pKa values from [1].

| PDB identifier | Emboss pKa set | | pKa set by Grimsley et al 2009 [1] | |
| --- | --- | --- | --- | --- |
|  | IER | pI | IER | pI |
| 1A7U | 0.4 | 4.5 | 0.5 | 4.4 |
| 1AKN | 0.9 | 5.7 | 1.1 | 5.7 |
| 1BN6 | 0.4 | 4.9 | 0.5 | 4.8 |
| 1CQW | 0.4 | 4.9 | 0.5 | 4.8 |
| 1CRL | 0.2 | 4.6 | 0.4 | 4.4 |
| 1CUB | 4.5 | 7.5 | 4.6 | 7.2 |
| 1EVE | 0.8 | 6.2 | 0.8 | 6.2 |
| 1EVQ | 0.5 | 4.9 | 0.6 | 4.9 |
| 1EXW | 1.9 | 6.8 | 1.6 | 6.8 |
| 1GKK | 0.9 | 6.5 | 0.8 | 6.5 |
| 1I6W | 1.1 | 9.7 | 1 | 9.7 |
| 1IUO | 0.6 | 4.9 | 0.7 | 4.9 |
| 1J1I | 1.2 | 6.2 | 1.3 | 6.3 |
| 1JKM | 0.2 | 4.6 | 0.3 | 4.5 |
| 1JU3 | 0.2 | 4.5 | 0.3 | 4.2 |
| 1KU0 | 1.3 | 6.8 | 1.1 | 6.9 |
| 1L7R | 0.2 | 4.4 | 0.2 | 4.2 |
| 1LGY | 1.7 | 9.2 | 1.8 | 9.2 |
| 1MAA | 1 | 6 | 1 | 6.1 |
| 1ODS | 1.2 | 5.7 | 1.2 | 5.7 |
| 1OXM | 4.5 | 8.8 | 4.7 | 8.4 |
| 1QJ4 | 0.8 | 5.1 | 1 | 5.1 |
| 1TCA | 4.3 | 6 | 4.5 | 5.9 |
| 1XZA | 4.5 | 8.8 | 4.7 | 8.4 |
| 2CUT | 4.5 | 8.8 | 4.7 | 8.4 |

**References**

1. Grimsley GR. Scholtz JM. Pace CN (2009) A summary of the measured pK values of the ionizable groups in folded proteins. Protein Science 18: 247-251.

2. Dolinsky TJ. Czodrowski P. Li H. Nielsen JE. Jensen JH. et al. (2007) PDB2PQR: expanding and upgrading automated preparation of biomolecular structures for molecular simulations. Nucleic Acids Res 35: W522-525.
